# Molecular diagnosis of autochthonous human anaplasmosis in Austria – an infectious diseases case report

**DOI:** 10.1186/s12879-020-04993-w

**Published:** 2020-04-19

**Authors:** Wolfgang Hoepler, Mateusz Markowicz, Anna-Margarita Schoetta, Alexander Zoufaly, Gerold Stanek, Christoph Wenisch

**Affiliations:** 1 4th Medical Department with Infectious Diseases and Tropical Medicine, Sozialmedizinisches Zentrum Süd - Kaiser-Franz-Josef-Spital mit Gottfried von Preyer’schem Kinderspital, KFJ-Spital, Kundratstrasse 3, 1100 Vienna, Austria; 2 Institute for Hygiene and Applied Immunology, Center for Pathophysiology, Infectiology and Immunology, Medical University of Vienna, Kinderspitalgasse 15, 1090 Vienna, Austria

**Keywords:** Infectious diseases, *Anaplasma phagocytophilum*, Molecular diagnosis, Tick-borne diseases in Western Europe

## Abstract

**Background:**

The diagnosis of human anaplasmosis remains elusive and is probably often missed. This case report highlights the efficacy of molecular diagnostic techniques.

**Case presentation:**

We would like to report the case of a 74-year-old man who was admitted to hospital because of a high fever, marked chills, transient diplopic images and vertigo, 6 weeks after multiple tick bites. The laboratory results showed mild anemia, marked thrombocytopenia and leukopenia and a moderately elevated C-reactive protein. The initial serology seemed to indicate an active infection with *Borrelia* spp., and *Anaplasma phagocytophilum* was detected in peripheral blood by polymerase chain reaction (PCR) and subsequent sequencing. The patient received intravenous ceftriaxone for 14 days and oral doxycycline for 4 weeks and made a fast and complete recovery.

**Conclusions:**

While human anaplasmosis has been reported very rarely in Austria, it should be considered as a differential diagnosis in febrile patients with low leukocyte and platelet counts with elevated levels of C-reactive protein after exposure to tick bites. Molecular detection of *A. phagocytophilum* is the technique of choice allowing rapid and reliable diagnosis.

## Background

The identification of pathogens which are not detectable by commercial blood culture systems is highly challenging, even more so when the responsible agent only rarely causes diseases in humans and is, therefore, frequently overlooked. This case report highlights the importance of broadening the diagnostic horizon and making use of modern diagnostic techniques, such as specific polymerase chain reaction as well as attention to the medical history and epidemiological awareness of tick-transmitted infections.

## Case presentation

The patient was a 74-year-old man in an excellent physical condition with an unremarkable past medical history apart from arterial hypertension which was well-controlled under regular medication with doxazosin, amlodipin and candesartan. Furthermore, he was on primary cardiovascular prophylaxis with low-dose acetylic salicylic acid.

He was an Austrian citizen with no recent travel activity abroad, but he liked spending time outdoors.

In August 2018, while on vacation in the lake district of Upper Austria, the patient suddenly collapsed (without loss of consciousness) while driving a car, and developed vertigo, diplopic images and profuse vomiting. Due to the abrupt onset of neurological symptoms, he was urgently transported by a helicopter of the emergency medical services to a nearby stroke unit.

The patient arrived at the hospital in a stable condition and with his neurological symptoms gone, but he complained about reduced appetite and having felt generally unwell and running fever spikes up to 38.8 °C for at least 10 days.

The physical and in-depth neurological examinations were unremarkable. Intracranial bleeding, sinus vein thrombosis and ischemic stroke were immediately excluded by cerebral computed tomography (CT) including CT-angiography and magnetic resonance imaging.

Three sets of blood cultures were drawn, and the initial laboratory investigations revealed mild anemia (haemoglobin 10.8 g/dL), thrombocytopenia (128 G/L) and elevated C-reactive protein (49 mg/L). The leukocyte count (4.4 G/L), the differential count and liver function tests were normal.

Chest X ray, urine analysis and duplex sonography of the cerebral arteries were also unremarkable.

Because of potential bleeding risk due to the intake of acetylic salicylic acid and because of patient refusal, no lumbar puncture was performed.

The patient was put on empiric antibiotic therapy with intravenous ceftriaxone 2 g /day and received supportive treatment with intravenous rehydration and paracetamol.

However, he was still severely ill and continued having a high temperature (up to 39.5 °C) and dramatic chills, and the leukocyte (3 G/L) and platelet (72 G/L) counts showed a marked decrease, while CRP rose up to 72 mg/L.

Therefore, on the third hospital day, three more sets of blood cultures were collected and therapy was escalated to intravenous piperacillin/tazobactam 4.5 g tid.

Transthoracic echocardiography was performed twice and revealed mild aortic and mitral regurgitation without any signs of endocarditis or pericarditis.

Diagnostic work-up revealed positive cytomegalovirus IgM with negative IgG antibodies, positive IgM and IgG antibodies for varicella zoster virus, positive Epstein-Barr virus IgG antibodies with negative IgM, and positive *Borrelia burgdorferi* sensu lato (sl) ELISA IgM, but a negative western blot.

On the fourth day, the patient was transferred to the intermediate care unit of our department of infectious diseases in Vienna.

Here, the patient presented with ongoing high fever and marked chills, but was otherwise in a stable condition.

His dental status was unremarkable, his last check-up had taken place 2 months earlier with no intervention or dental hygiene; he had no pets, and there had been no other sick persons in his surroundings; he had not traveled into tropical regions or abroad in the last few years. He had noticed several tick bites about 6 weeks earlier, one of them possibly showing a halo sign.

Endocarditis was definitively excluded by an unremarkable transesophageal echocardiography, repeatedly negative blood cultures and a negative broad-spectrum PCR (Septifast, Roche Diagnostics, Switzerland).

A CT scan of the chest and abdomen showed old splenic infarcts and a lesion (diameter 1.2 cm) in the pancreatic head, which later turned out to be a benign intrapancreatic cyst (diagnostic modalities were MRI and endo-sonography enabling histological work-up).

We continued antibiotic therapy with ceftriaxone, and added empiric treatment with intravenous doxycycline 2 × 100 mg for possible atypical bacterial pathogens and intravenous acyclovir 3 × 10 mg/kg for possible herpes virus encephalitis, which could not be definitely excluded at this stage without a lumbar puncture.

Leptospirosis, mycoplasma infection, babesiosis, tick-borne encephalitis, West Nile virus encephalitis, primary infection with cytomegalovirus, herpes simplex virus encephalitis and infection with parvovirus B19 were ruled out by negative serologic tests.

The patient went on having high fever spikes and chills and developed marked thrombocytopenia (minimum 49 G/L, but no bleeding occurred), leukopenia (2.4 G/L), more pronounced anaemia (8.8 g/dL) and CRP elevation up to 117 mg/L. Also, there was a transient, self-limiting rise in transaminases starting on the 8th day of illness (maximum AST 115/ ALT 89 U/l) as well as in lactate dehydrogenase (maximum 304 U/l).

**Figure 1:**
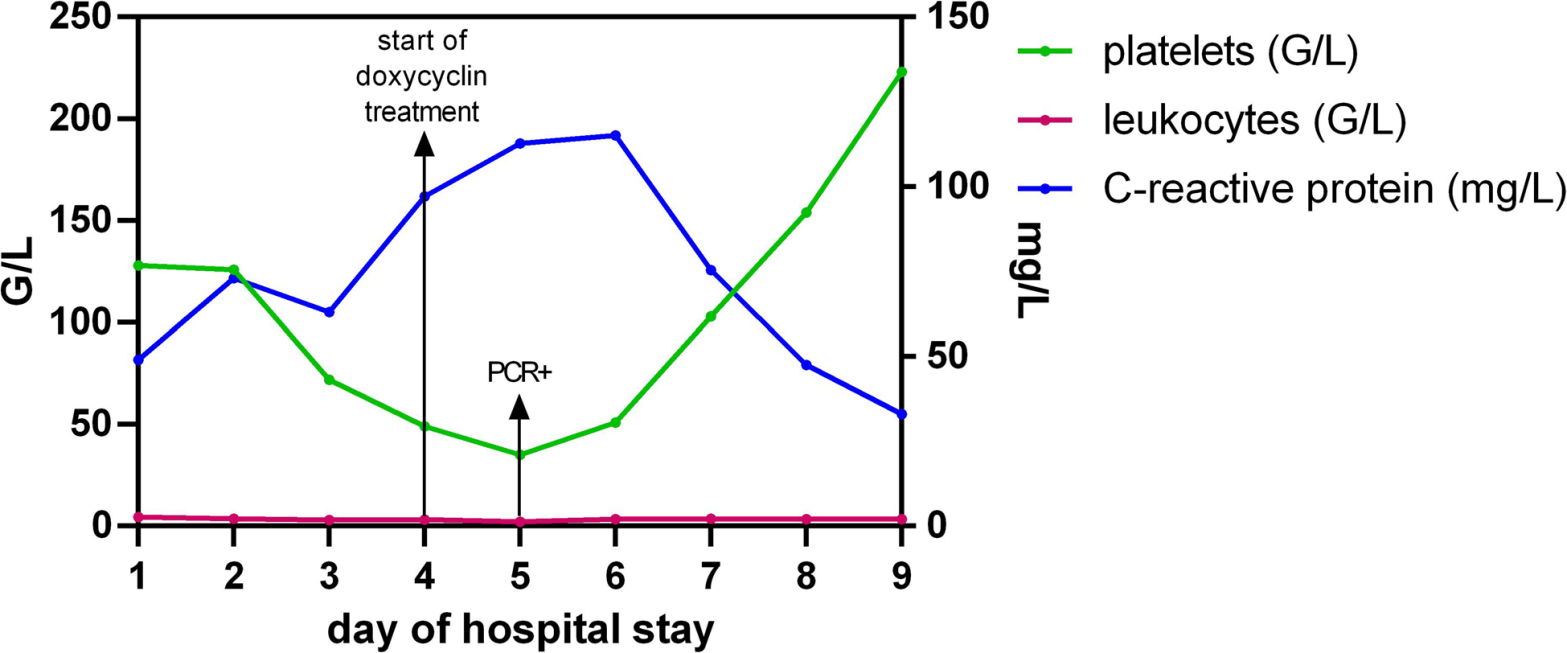
Fig. 1 Laboratory results

Laboratory results are shown in Fig. 1.

Two weeks after the onset of symptoms, samples were sent to the reference laboratory at the Medical University of Vienna for molecular and serologic testing as infections with *A. phagocytophilum* and *B. burgdorferi* sl were suspected. DNA from 2 ml EDTA blood was extracted using the QuickGene DNA whole blood kit L (Fujifilm) and the automated isolation system QuickGene-610 L (Fujifilm). Realtime PCR was based on a fragment of the 16S rRNA gene [1] and performed with an in-house PCR mastermix. The PCR was positive and further on confirmed by a conventional PCR followed by sequencing. This PCR also targeted the 16S rRNA gene and was conducted using the Phire Hot Start II Polymerase kit (Fisher Scientific, Vienna, Austria) and primers 16S8FE [2] and Ehr-R [3]. All PCRs were carried out according to molecular biological rules including appropriate controls. The amplicon was purified from agarose gel (Qiagen Gel Extraction Kit, Qiagen, Hilden, Germany) and sent in for bi-directional sequencing (Microsynth, Vienna, Austria). The presence of *A. phagocytophilum* was confirmed by comparing the consensus sequence to data available at the NCBI (https://blast.ncbi.nlm.nih.gov/Blast.cgi) yielding a 100% identity to various *A. phagocytophilum* strains.

An immunofluorescence assay (IFA) for *A. phagocytophilum* IgG antibodies (Focus Diagnostics, Cypress, California, USA) was also positive at a titre of 1:512 (positive cut-off ≥1:64). Additionally, IgM antibodies against *B. burgdorferi* sl were found both by ELISA and immunoblot, but no specific IgG antibodies were detected (ELISA and Lineblot, Euroimmun, Lübeck, Germany). The conflicting results with the initial serological results may be explained by the fact that they were performed by different laboratories and different thresholds were applied.

Thus, the definitive diagnosis of *A. phagocytophilum* infection was established, and acyclovir was stopped. The patient defervesced 24 h after the start of doxycycline, and remained afebrile henceforth.

As concurrent neuroborreliosis could not be safely excluded at that point, the patient was treated with ceftriaxone for a total of 14 days, after which he could be discharged from hospital, plus oral doxycycline (100 mg bid) for a total of 4 weeks (for possible Lyme neuroborreliosis). Ceftriaxone may have been over-treatment, since doxycycline is also effective in Lyme neuroborreliosis. The patient made a rapid and full recovery, and thrombocytopenia and leukopenia resolved within 4 days and 2 days, respectively, after initiation of doxycycline therapy.

At a check-up 3 weeks later, serologic testing of a paired serum sample was done at the reference laboratory. The *Anaplasma* IgG IFA titre showed a twofold increase (testing for IgM was not performed). Moreover, there was a decline of the ELISA *Borrelia* IgM levels from > 200 U/ml to 121 U/ml (positive cut-off > = 22 U/ml) confirmed by immunoblot in both cases, whereas the IgG ELISA level was negative. These results suggest a recent infection with *B. burgdorferi* s.l. However, a conclusion about the exact time point of the infection cannot be drawn.

The patient himself reported no side-effects whatsoever of the medication used, assured us of 100% adherence and claimed to have completely regained his normal health status.

## Discussion and conclusions

*Anaplasma phagocytophilum* is an obligate intracellular gram-negative bacterium which was identified as a human pathogen in the 1990s and first described by Chen et al. in 1994 [4]. The first case in Europe was reported from Slovenia in 1997 [5].

Deer and the white-footed mouse are considered the principal hosts [6], and the vectors are ixodid tick species, in Europe mainly *Ixodes ricinus* [7].

As can be inferred from sero-prevalence studies, infection by *Anaplasma* spp. is underreported [8], partly because of its unspecific presentation and a significant number of asymptomatic or subclinical courses.

Moreover, the diagnosis of anaplasmosis remains highly challenging as cultivation of Anaplasmataceae is extremely difficult and time-consuming [9], and suitable target sequences of the organism may not be included in every conventional broad spectrum PCR assay [10]. Therefore, species-specific nucleic acid amplification has proven to be the most reliable method for the detection of Anaplasmataceae, with the caveat that specificity and sensitivity are highly variable depending on the assays used [11].

The patient presented here displayed many of the characteristic clinical features of anaplasmosis, such as fever, chills, malaise, marked leukopenia and thrombocytopenia [12].

While the incubation period is usually in the range of one to 2 weeks [13], more protracted clinical courses, such as in our patient, have been described [14].

*Anaplasma* spp. are universally susceptible to doxycycline [15], which is regarded as the antibiotic of choice, due to its reliable activity both against *Anaplasma* spp. and possible co-infection with *Borrelia burgdorferi* sensu lato.

Only a handful of cases of human granulocytic anaplasmosis acquired autochthonously in Austria have been described before [16–18].

Anaplasmosis is often a benign and self-limiting illness [19]. However, since fatal outcome has been reported [20], thoughtful medical history and immediate medical action is pivotal in a febrile patient with leuko – and thrombocytopenia and elevated levels of CRP [21] reporting a tick bite. This may ultimately save the patient’s life and usually leads to a rapid and full recovery.

In our patient, there was serologic evidence of a recent infection with *B. burgdoferi* s.l. although the exact time point of this event is difficult to establish. The patient reported many tick bites in the past, one of them followed by a halo sign – possibly an erythema migrans.

This case report highlights the need to always take a thorough medical history in complicated cases, to consider rare (but endemic) infections and to apply modern diagnostic techniques, such as specific polymerase chain reaction.

## Data Availability

All available medical data are safely stored at the hospital and can be provided on request.

## Abbreviations

bid: Twice daily
CRP: C-reactive protein
CT: Computed tomography
°C: Degrees Celsius
EDTA: Ethylenediamine tetraacetic acid
ELISA: Enzyme-linked immunosorbent assay
g/dL: Gram/deciliter
G: Giga
g: Gram
IgM/G: Immunoglobulin M/G
L: Liter
mg: Milligram
MRI: Magnetic resonance imaging
PCR: Polymerase chain reaction
spp-: Species
tid: Three times daily
